# Txnip/Trx Is a Potential Element in Regulating O-GlcNAc Modification in Photoreceptors to Alleviate Diabetic Retinopathy

**DOI:** 10.3390/ijms26115369

**Published:** 2025-06-04

**Authors:** Laraib Imdad, Shengnan Xu, Yulang Meng, Kaimin Bao, Wenkang Dong, Xuanya Yin, Yujie Tong, Wei Zhang, Xiang Ren, Li Kong

**Affiliations:** 1 Department of Histology and Embryology, College of Basic Medicine, Dalian Medical University, Dalian 116044, China; 2 Key Laboratory of Reproductive and Developmental Biology, Dalian Medical University, Dalian 116044, China

**Keywords:** diabetic retinopathy, O-GlcNAcylation modification, photoreceptors, thioredoxin, thioredoxin-interacting protein

## Abstract

Hyperglycemia is a key factor in diabetic retinopathy which leads to blindness. O-linked-N-acetylglucosamine (O-GlcNAc) modification changes are linked to various diseases, including diabetic retinopathy. This research aims to study the roles of Txnip and Trx in influencing O-GlcNAc in photoreceptor cells during diabetic retinopathy. A diabetic mouse model and 661w cells, after exposure to high glucose, were employed as models. H&E staining and ERG were used to evaluate the morphology and function of the retina, respectively. Western blotting was used to analyze protein expression, a TUNEL assay was used to measure apoptosis, and a co-immunoprecipitation (CO-IP) assay was used to detect the interactions of protein. In diabetic mice, electroretinogram (ERG) amplitude wave, retinal thickness, and body weight decreased. Glial fibrillary acidic protein (GFAP), Iba1 expression, and blood glucose level increased. In vitro, the percentage of apoptotic cell, Bax, and caspase3 levels increased, and Bcl2 decreased in 661w cells under high-glucose conditions. Moreover, Txnip expression was upregulated, while Trx was downregulated. Additionally, a Western blot analysis revealed that high-glucose exposure led to increased O-GlcNAc modification both in vivo and in vitro. The CO-IP results show that Txnip interacted with O-GlcNAc modifications. S-opsin expression was significantly downregulated in vitro under high-glucose conditions. Knockdown Txnip or upregulation Trx could reverse or delay apoptosis in 661w cells under hyperglycemic conditions. Txnip/Trx is a potential element in regulating photoreceptor apoptosis in diabetic retinopathy. The underlying mechanism is linked to regulation of O-GlcNAc modification in photoreceptor cells in DR.

## 1. Introduction

Diabetes mellitus (DM) is associated with a range of complications, including cardiovascular diseases, nephropathy, and retinopathy, which significantly contribute to morbidity and mortality in diabetic patients [1]. Diabetic retinopathy (DR) is a microvascular complication of diabetes characterized by a progression of pathological pathways, including the formation of microaneurysms, vascular hyperpermeability, and retinal ischemia. Several studies have reported that pathological changes, including microglial activation and neuronal injury, can occur in the retinal neurovascular unit (NVU) prior to the onset of overt vascular injury in DR. These findings emphasize the importance of early events in the disease process [2]. Therefore, it is essential to find some new manner or target molecule to prevent DR during the early stage. In our previous research, we have shown that preventing apoptosis in 661w photoreceptor cells in a hyperglycemic state, which is essential for maintaining photoreceptor structure, function, and microenvironment stability, can significantly delay the onset and progression of DR in vitro [3].

Müller cells, which are the principle glial cells in the retina [4], along with microglia, play a key role as a regulator of homeostasis and undergo reactive transformation in DR. Microglia play a crucial role in retinal homeostasis, contributing to normal retinal growth, neurogenesis, synaptic pruning, and vascular development. They interact with retinal glial cells by secreting growth factor and neuroprotective mediators [5]. Several studies have reported that microglial activation, characterized by morphological changes, can precede neuronal apoptosis and the activation of other glial cells in diabetic retina, suggesting a key role for microglia in the initiation of the neuroinflammatory process [6]. Activated microglia contribute to DR progression by releasing pro-inflammatory cytokines, vascular endothelial growth factor (VEGF), and reactive oxygen species (ROS), which disrupt the blood–retinal barrier (BRB) and promote both vascular leakage and neuroinflammation. Furthermore, dysfunctional Müller cells fail to provide adequate nutrition and antioxidants to photoreceptor cells, leading to mitochondrial dysfunction, apoptosis, and visual impairment [7,8]. Understanding the detrimental microenvironmental effects mediated by Müller and microglial cells on photoreceptors is crucial for developing novel therapeutic strategies to preserve retinal integrity in DR [9].

Multiple factors contribute to the pathogenesis of DR, including the accumulation of advanced glycation end products (AGEs), increased oxidative stress, activation of polyol pathway, and protein kinase C activation [3,10]. O-GlcNAcylation is a unique post-translational modification (PTM) present in both cytoplasmic and nuclear proteins, and it is regulated by O-GlcNAc transferase (OGT) and O-GlcNAcase (OGA) [11]. This post-translational modification is regulated by nutrient availability, playing a critical role in cellular stress responses [12]. In the context of DR, hyperglycemia induces irregular O-GlcNAcylation, disrupting normal cellular signaling and homeostasis [13]. This modification affects proteins involved in stress response [14] and apoptosis [15], all of which are pathways implicated in DR progression.

Thioredoxin (Trx), a 12 kDa redox protein with a conserved dithiol/disulfide active site, is ubiquitously expressed in mammalian tissues. Thioredoxin-interacting protein (Txnip), initially identified in vitamin-D3-upregulated protein 1 (VDUP1), is recognized as a protein that binds to Trx, thereby inhibiting Trx redox functions. Txnip has been shown to play an important role in glucose regulation and lipid metabolism [16]. Disruption to O-GlcNAcylation contributes to the upregulation of Txnip, a key mediator of oxidative stress and mitophagy, thereby contributing to the pathogenesis of DR [17].

Therefore, we employed 661w, Müller, and BV2 cell lines under high-glucose treatment to induce the DR model in vitro to explore this mechanism, aiming to provide valuable insight into potential therapeutic targets for DR. While both O-GlcNAcylation and the Txnip/Trx axis are linked to DR, their direct interplay in photoreceptors remains unclear. This study is the first to explore their crosstalk under hyperglycemic conditions.

## 2. Results

### 2.1. Diabetes-Induced Retinal Damage in Mice

**Figure 1:**
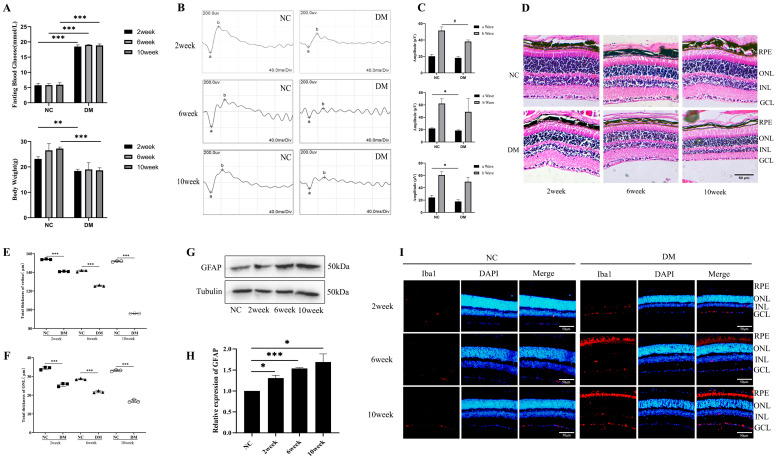
Diabetes-induced retinal damage in vivo: (**A**) body weight and fasting blood glucose level in diabetic mice; (**B**,**C**) retinal function as measured by electroretinography in diabetic mice, where the letter “a” represents the peak of the “a wave”, and “b” represents the peak of the “b” wave; (**D**) retinal morphology of the diabetic mice as examined by HE staining, where the scale bar is 50 μm; (**E**,**F**) total retinal thicknesses and ONL measured in diabetic mice; (**G**,**H**) Western blot was used to investigate the expression of GFAP in diabetic mice at 2, 6, and 10 weeks; (**I**) immunofluorescence staining method was used to detect the expression of iba1 activation at 2, 6, and 10 weeks in diabetic mice. The statistical data are expressed as the mean ± SD. * *p* < 0.05; ** *p* < 0.01; *** *p* < 0.001. # *p* < 0.05.

In Figure 1A, a significant reduction in body weight in the diabetic group compared with the control mice is demonstrated. Similarly, a significant elevation in fasting blood glucose levels was observed in the diabetic mice compared with the controls. At 2 weeks, the diabetic mice exhibited 18.13 ± 0.40 mmol/L compared with 5.47 ± 0.55 mmol/L in the controls (*p* < 0.001). At 6 weeks, the diabetic mice showed 19.04 ± 0.12 mmol/L compared with 5.80 ± 0.51 mmol/L in the controls (*p* < 0.001). By 10 weeks, the blood glucose in the diabetic mice remained elevated at 18.78 ± 0.54 mmol/L compared with 5.60 ± 0.59 mmol/L in the controls (*p* < 0.001). The ERG waveform (Figure 1B,C) results show that the peak values of the a and b waves of the diabetic mice decreased compared with those of the control mice. The a-wave amplitude was lower in the diabetic group, at 18.27 ± 1.61 µV, compared with the control group, at 20.27 ± 2.12 µV (*p* < 0.05), and the b-wave amplitude also significantly decreased in the diabetic mice, at 38.47 ± 1.84 µV, compared with the control, at 51.47 ± 4.47 µV (*p* < 0.05 and *p* < 0.05). Moreover, the total retinal thickness and the outer nuclear layer (ONL) were also reduced in the diabetic mice. At 2 weeks, the diabetic mice exhibited 25.58 ± 0.88 µm compared with 34.32 ± 0.72 µm in the normal control, at 6 weeks they showed 21.78 ± 0.51 µm, and at 10 weeks they exhibited 16.49 ± 0.67 µm compared with 28.55 ± 0.33 µm and 33.11 ± 0.34 µm in the normal controls (Figure 1D–F). The statistical analysis indicated significant degradation in the retinal parameters in the diabetic mice compared with the normal controls (*p* < 0.001). The expression of GFAP increased in the diabetic mice (*p* < 0.05 and *p* < 0.001) (Figure 1G,H), and the immunofluorescence results showed that the expression of Iba1 (Red) (Figure 1I) also increased in the diabetic mice compared with the control mice. The blue color indicates DAPI.

### 2.2. Diabetes-Induced Retinal Cell Damage Is Associated with Alterations in O-GlcNAc Modification

**Figure 2:**
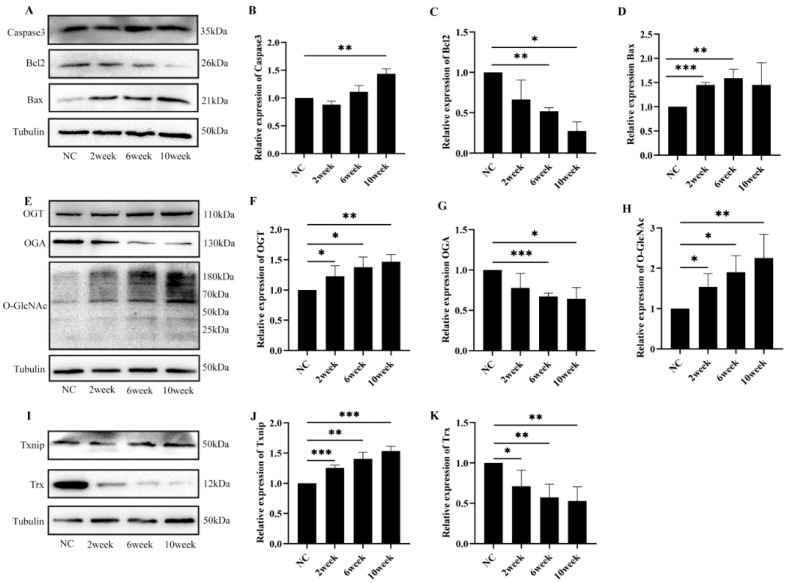
Diabetes-induced retinal cell damage is associated with alterations in O-GlcNAc modification: (**A**) Western blot analysis was employed to assess the protein expression levels of apoptotic-related protein in diabetic mice; (**B**) Western blot analysis statistics for caspase3; (**C**) Western blot analysis of Bcl-2; (**D**) Western blot analysis of Bax; (**E**) O-GlcNAc modification level in diabetic mice evaluated by Western blot; Western blot band analysis of OGT (**F**), OGA (**G**), and O-GlcNAc (**H**); (**I**) effects of hyperglycemia on increased Txnip levels and decreased Trx levels in diabetic mice, as detected by Western blot; statistical analysis of Western blots for Txnip (**J**) and Trx (**K**) expression. The statistical data are expressed as the mean ± SD. * *p* < 0.05; ** *p* < 0.01; *** *p* < 0.001.

The results show a significant downregulation of the pro-survival protein Bcl-2, while the pro-apoptotic protein Bax and caspase3 exhibited marked increases comparatively in the diabetic mice (*p* < 0.05 and *p* < 0.01) (Figure 2A–D). Western blot analysis revealed significant upregulation of O-GlcNAc and OGT protein expression in the diabetic mice compared with the control group. Conversely, OGA expression exhibited a marked downregulation (*p* < 0.05, *p* < 0.01, and *p* < 0.001) (Figure 2E–H). In addition, in the diabetic mice there was a progressive decline in Trx protein levels, while Txnip protein exhibited a corresponding increase (*p* < 0.05, *p* < 0.01, and *p* < 0.001) (Figure 2I–K).

### 2.3. High-Glucose-Induced 661w Cell Apoptosis Is Related to O-GlcNAc Modification

**Figure 3:**
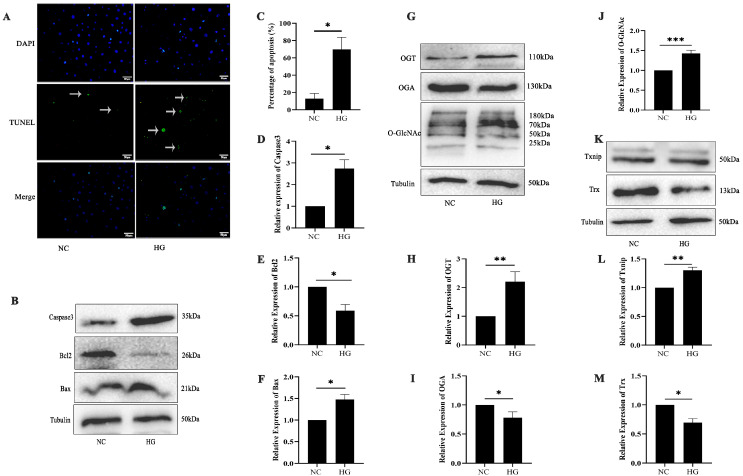
Diabetes-driven photoreceptor cell damage is associated with O-GlcNAc modification changes in vitro. (**A**) High-glucose treatment induced apoptosis in 661w cells, as observed in a TUNEL assay. Cells undergoing apoptosis were identified by green fluorescent TUNEL staining. (**B**) Western blot analysis detected the expression of caspase3, Bcl2, and Bax after treatment with high glucose in 661w cells. (**C**) Statistical analysis of the TUNEL assay. Statistical analysis of caspase3 (**D**), Bcl2 (**E**), and Bax (**F**). (**G**) Western blot analysis was employed to assess O-GlcNAc, OGT, and OGA expression in 661w cells following high-glucose treatment. Statistical analysis of OGT (**H**), OGA (**I**), and O-GlcNAc (**J**). (**K**) Significant alterations in Txnip and Trx protein levels after treatment with high glucose in 661w cells, as confirmed by Western blot. (**L**) Statistical analysis of Txnip. (**M**) Statistical analysis of Trx. The statistical data are expressed as the mean ± SD. * *p* < 0.05; ** *p* < 0.01; *** *p* < 0.001.

TUNEL staining revealed an increased apoptosis in 661w cells following high-glucose treatment (*p* < 0.05) (Figure 3A,C). 661w cells were initially treated with 50 mM high-glucose media for 24 h. Significant upregulation of Bax and caspase3 and downregulation of Bcl-2 protein expression were observed, which suggest a potential shift toward a pro-apoptotic state (*p* < 0.05) (Figure 3B,D–F). High-glucose exposure led to coordinated increases in O-GlcNAc and OGT protein levels, while OGA expression displayed a corresponding decrease compared with the controls (*p* < 0.05, *p* < 0.01, and *p* < 0.001) (Figure 3H–J). Exposure to high glucose levels resulted in a significant upregulation of Txnip protein expression, accompanied by an approximately 32% reduction in Trx protein levels in the 661w cells (*p* < 0.05 and *p* < 0.01) (Figure 3K–M).

### 2.4. Effects of Txnip on High-Glucose-Induced Photoreceptor Cells’ Apoptosis with O-GlcNAc Modification Alterations In Vitro

**Figure 4:**
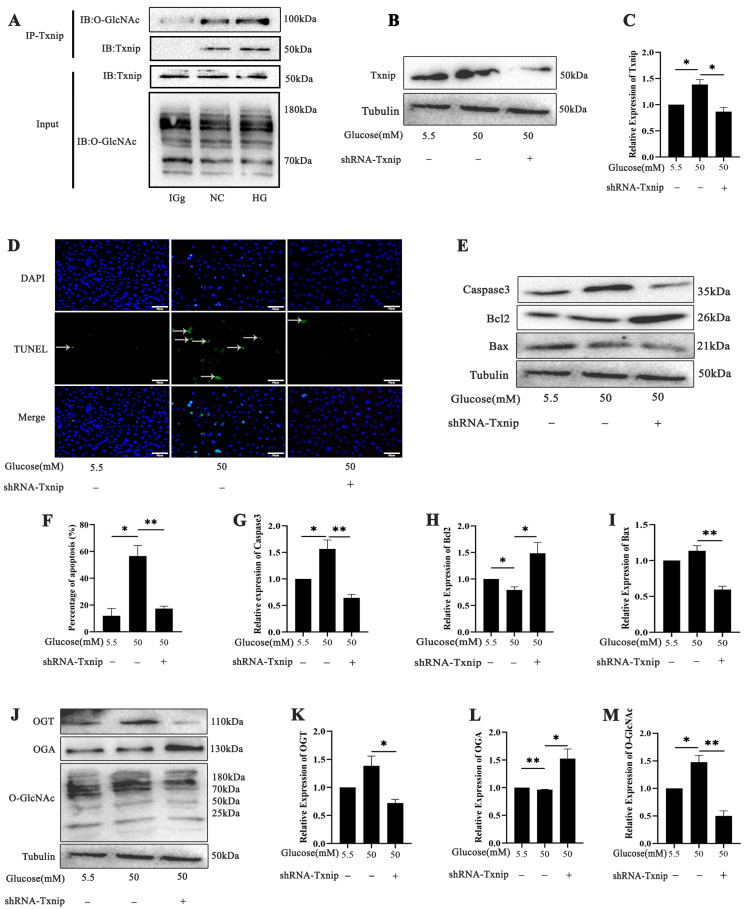
Effect of Txnip on high-glucose-induced 661w cells’ apoptosis with O-GlcNAc modification alterations. (**A**) The interaction of Txnip and O-GlcNAc modification changes in 661w cells after high-glucose treatment was examined using a CO-IP assay. (**B**,**C**) Western blot was used to evaluate the expression of Txnip in 661w cells under high-glucose conditions with and without shTxnip treatment and statistical analysis of Txnip expression. (**D**,**F**) The effect of shTxnip on 661w cells under high-glucose conditions was analyzed by TUNEL assay and statistical data analysis. TUNEL-positive cells marked by green fluorescence. (**E**) Western blot analysis of apoptotic protein expression and graphs of the statistical analyses of caspase3 (**G**), Bcl2 (**H**), and Bax (**I**) in 661w cells under high-glucose conditions with and without shTxnip treatment. (**J**) OGT, OGA, and O-GlcNAc expression was evaluated by Western blot, (**K**–**M**) and their bands were statistically analyzed. The statistical data are expressed as the mean ± SD. * *p* < 0.05; ** *p* < 0.01.

We verified the relationship between O-GlcNAc and Txnip through a CO-IP assay to confirm the Txnip-O-GlcNAc modification in the 661w cells (Figure 4A). The shRNA Txnip plasmid was transfected into 661w cells to achieve gene silencing (Figure 4B,C). In the high-glucose-treated group, the expression of Txnip protein was significantly elevated, as evidenced by the intensity of the corresponding band. Conversely, in the shTxnip-treated group, the Txnip protein band intensity was markedly reduced, indicating successful knockdown of Txnip in the 661w cells (*p* < 0.05 and *p* < 0.01). A TUNEL assay was performed to assess the level of apoptosis in 661w cells under high-glucose conditions. The TUNEL results and bar graph demonstrate a significant increase in apoptotic cells in the high-glucose-treated group. However, for 661w cells treated with shTxnip under high-glucose conditions, the number of apoptotic cells was notably reduced (Figure 4D,F). The expression levels of the apoptotic-related proteins, including caspase3, Bcl-2, and Bax, were analyzed to evaluate the impact of high-glucose treatment and shTxnip intervention. In the high-glucose-treated group, Bax and caspase3 levels significantly increased, while Bcl-2, an anti-apoptotic protein, was markedly reduced, indicating enhanced apoptotic activity under hyperglycemic conditions (Figure 4E,G–I) (*p* < 0.05 and *p* < 0.01). The expression levels of O-GlcNAc and OGT were markedly elevated in the high-glucose-treated group, consistent with the hyperglycemic conditions promoting increased protein O-GlcNAcylation while OGA was reduced. However, in the shTxnip-treated group, the expression of O-GlcNAc, OGT, and OGA was significantly altered after shTxnip treatment under high-glucose conditions (Figure 4J–M) (*p* < 0.05 and *p* < 0.01).

### 2.5. Effects of Trx Overexpression from Müller-Cell-Conditioned Media on O-GlcNAc Modification Change in 661w Cells in a Hyperglycemic State

**Figure 5:**
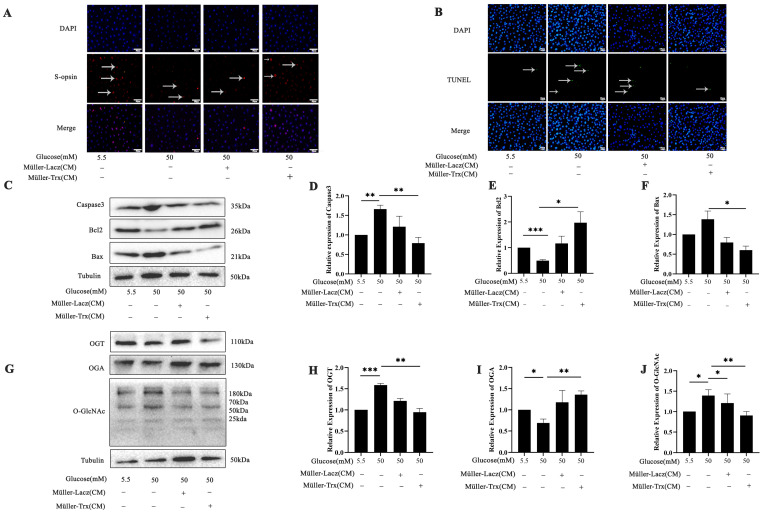
Trx-enriched, conditioned media from Müller cells affected O-GlcNAc modification in 661w cells during DR in vitro. (**A**) Immunofluorescence of S-opsin expression detected in 661w cells treated with and without Trx-enriched, conditioned media from Müller cells under high-glucose conditions. (**B**) TUNEL assay was used to assess apoptosis in 661w cells following treatment. Apoptotic cells were identified as TUNEL-positive, exhibiting green fluorescence; scale bar 50 μm. (**C**) Western blot analysis was employed to evaluate the expression of apoptosis-related proteins caspase3, Bcl-2, and Bax in 661w cells following treatment. (**D**) Statistical analysis of caspase3. (**E**) Statistical analysis of Bcl2. (**F**) Statistical analysis of Bax. (**G**) Western blot analysis of OGT, OGA, and O-GlcNAc expression levels in 661w cells treated with Trx-enriched media from Müller cells under high-glucose conditions; OGT (**H**), OGA (**I**), and O-GlcNAc (**J**). The statistical data are expressed as the mean ± SD. * *p* < 0.05; ** *p* < 0.01; *** *p* < 0.001.

To investigate the influence of the microenvironment on photoreceptor cells under hyperglycemia in vitro, we employed conditioned medium derived from Müller-Lacz and Müller-Trx cells, which are Müller cell lines that are stably overexpressing Lacz and Trx. Immunofluorescence was used to evaluate the expression of S-opsin in 661w cells under high-glucose conditions, and the results demonstrate the distinct difference in S-opsin levels between the Trx-enriched, conditioned-media-treated group and the high-glucose group (Figure 5A). The TUNEL staining revealed a significant decrease in the apoptotic fraction of 661w cells treated with Trx-enriched, conditioned media from Müller cells compared with only high-glucose-treated cells (Figure 5B). Western blot analysis was performed to assess the apoptotic levels in 661w cells after conditioned media treatment. Specifically, anti-apoptotic Bcl-2 protein levels significantly increased, while pro-apoptotic Bax and caspase3 protein levels significantly decreased in the conditioned-media-treated group (Figure 5C–F) (*p* < 0.05, *p* < 0.01, and *p* < 0.001). Notably, O-GlcNAc and OGT levels also significantly decreased, while OGA expression displayed a significant increase in the conditioned-media-treated group (Figure 5G–J) (*p* < 0.05, *p* < 0.01, and *p* < 0.001).

### 2.6. Effects of Trx Overexpression from BV2-Cell-Conditioned Media on O-GlcNAc Modification Change in 661w Cells in a Hyperglycemic State

**Figure 6:**
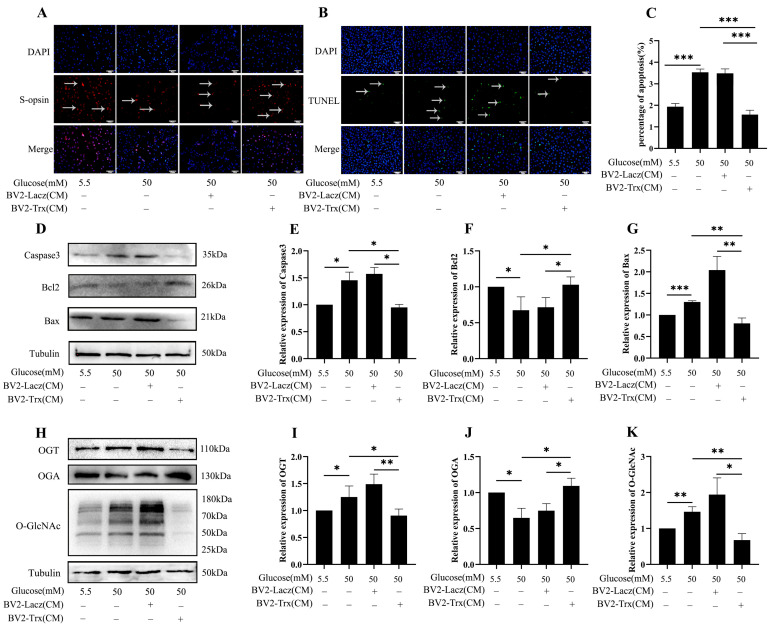
The protective effects of Trx-conditioned media from BV2 cells affected O-GlcNAc modification in 661w cells during DR in vitro. (**A**) The expression of S-opsin with Trx-enriched media from BV2 cells in 661w cells evaluated by immunofluorescence. (**B**) TUNEL staining identified apoptotic cells in treated 661w cells and TUNEL-positive cells were labeled by green fluorescence. Arrows highlighting TUNEL-positive cells are presented; scale bar is 50 μm. (**C**) Statistical analysis of the TUNEL. (**D**) Western blot analysis was performed to evaluate the expression of caspase3, Bcl2, and Bax. (**E**) Statistical analysis of caspase3. (**F**) Statistical analysis of Bcl2. (**G**) Statistical analysis of Bax. (**H**) Western blot analysis was assessed to evaluate the expression levels of OGT, OGA, and O-GlcNAc in 661w cells. Statistical analysis of the bands in OGT (**I**), OGA (**J**), and O-GlcNAc (**K**). The statistical data are expressed as the mean ± SD. * *p* < 0.05; ** *p* < 0.01; *** *p* < 0.001.

We also treated 661w cells with conditioned media from BV2-Lacz and BV2-Trx cells which BV2 cell lines that were stably overexpressing Lacz and Trx to investigate the effect of microglia on the microenvironment in DR. The result shows that elevated glucose levels decreased S-opsin expression in 661w cells (Figure 6A). However, Trx-enriched, conditioned media from BV2 cells restored S-opsin expression, indicating that Trx may possibly rescue 661w cell function under high-glucose conditions. TUNEL staining revealed a significant decrease in apoptotic cells in Trx-enriched, conditioned media from BV2 cells (Figure 6B,C) (*p* < 0.001). Western blot analysis revealed differential protein expression in 661w cells following treatment. Notably, treatment with Trx-enriched, conditioned media from BV2 cells resulted in the downregulation of the apoptotic proteins Bax and caspase3, while Bcl2 expression was upregulated (Figure 6D–G) (*p* < 0.05, *p* < 0.01, and *p* < 0.001). The OGT, O-GlcNAc, and OGA protein levels exhibited distinct changes (Figure 6H–K) (*p* < 0.05, *p <* 0.01, and *p* < 0.001).

## 3. Discussion

DM is characterized by a dysregulated nutrient metabolism resulting from insulin resistance and relative insulin deficiency. DR is a serious complication of diabetes, and a leading cause of vision loss in adults and especially in middle-aged and elderly populations in developed countries [3]. High glucose and hyperlipidemia are major contributors to the development of DR. In addition, many researchers have demonstrated that early pathological changes in DR development exhibit neurodegenerative characteristics [18,19,20]. This study demonstrates that high glucose induced retinal dysfunction and neurodegeneration in both in vivo and in vitro models.

However, it is important to note that diabetes mellitus presents in various forms (type 1, type 2, and gestational diabetes), and various animals are used in models employed for research. While our study utilized a streptozotocin (STZ)-induced diabetic model, which primarily mimics type 1 diabetes, to enhance the translational relevance of these findings, future research should include comparative studies employing human-derived retinal organoids and type 2 diabetes models (such as db/db mice).

O-GlcNAc signaling contributes to the pathogenesis of diabetes and has been implicated in DR. Our recent research shows that elevated O-GlcNAc levels play a crucial role in regulating apoptosis and are a key factor in the development of DR [3]. O-GlcNAcylation is a dynamic post-translational modification (PTM). This modification regulates protein by targeting serine and threonine residues. In contrast, O-GlcNAc modification is dynamically regulated in mammalian cells, with a single enzyme, OGT, catalyzing its addition and another enzyme, OGA, catalyzing its removal. Therefore, it is very important to understand the structure and productivity of OGT and OGA to unlock their therapeutic potential in DR, especially in light of emerging small-molecule inhibitors such as OSMI-1 (OGT inhibitor) and Thiamet-G (OGA inhibitor), which are being investigated in several illness models in recent research [21,22].

Previous studies have indicated that Txnip, by suppressing the antioxidant activity of thioredoxin, plays a significant role in the pathogenesis of diabetes [16]. The present research investigated the role of Txnip in the progression of DR and evaluated the potential therapeutic effect of Trx for its treatment. Our results confirm that Txnip expression is upregulated under high-glucose conditions both in vitro and in vivo. Prolonged hyperglycemia is a key driver in the development of DR and leads to hyper O-GlcNAcylation, which contributes to the pathogenesis of DR by inducing apoptosis in photoreceptor and glial cells under high-glucose conditions. Upregulating Txnip expression in photoreceptor cells can promote cellular dysfunction characterized by inhibited mitochondrial dysfunction, apoptosis, and increased ROS production in photoreceptor cells. To further elucidate the role of Txnip in DR, we designed and employed shRNA to knockdown Txnip expression. Our results demonstrate a significant difference between the high-glucose group and the shRNA group. In the high-glucose group, we observed a marked increase in Bax and caspase-3, accompanied by a significant decrease in Bcl-2 expression. Treatment with shRNA led to a significant decrease in Bax and caspase-3 levels while concomitantly increasing Bcl-2 protein levels in photoreceptor cells. The observed changes in O-GlcNAc, OGT, and OGA expression upon Txnip knockdown highlight a potential link between Txnip and the O-GlcNAcylation pathway, which warrants further investigation, including CRISPR-mediated Txnip knock-in or knockout models to fully understand its implications for retinal cell function and survival in DR.

However, the lack of reciprocal CO-IP experiments to confirm the observed protein–protein interaction is one limitation of our study. We used CO-IP to investigate the roles of Txnip and O-GlcNAc modification changes. However, reciprocal CO-IP might have reduced the potential antibody bias and provided additional confirmation. To strengthen the interaction evidence, future research should include reciprocal CO-IP or other validation techniques, such as proximity ligation analysis.

Extensive systematic reviews and meta-analyses indicate that DR affects one-third of individuals with diabetes worldwide. The prevalence of DR significantly increases after the age of 60, likely due to the longer duration of diabetes in this population [23,24,25]. The diabetic milieu adversely affects multiple components of the NVU, including the vascular endothelium, microglial cells, Müller cells, and immune cells, thereby contributing to the pathogenesis of DR [26]. At this stage, early dysfunction of the NVU disrupts communication between neurons and blood vessels, leading to impaired retinal blood flow. Concurrently, the blood–retinal barrier weakens, compromising its ability to maintain the protective microenvironment around the retina [27]. Early neurodegeneration in DR necessitates novel therapeutic targets. Müller cells, crucial for retinal homeostasis, become dysfunctional in diabetes, characterized by inflammation and apoptosis [28]. This reactive state contributes to neurodegeneration and exacerbates DR progression. Targeting Müller and microglial cell dysfunction may offer promising avenues for DR prevention and treatment [5]. Furthermore, our findings confirm that in vitro diabetic conditions induce Müller cell dysfunction, characterized by a reactive phenotype and upregulation of GFAP. Our Western blot results demonstrate that overexpression of Trx in Müller and microglial cells within the culture media provided protection by regulating O-GlcNAc modification to photoreceptor cells under high-glucose conditions. Microglia are resident immune cells in the retina. These cells are the key players in the inflammatory response associated with diabetic retinopathy. Hyperglycemia activates the microglia, contributing to neuroinflammation and oxidative stress [29]. Our findings suggest that Trx overexpression in microglia extends its protective effects beyond Müller cells, indicating a potential neuroprotective role for microglia in mitigating retinal damage under a stressful state. These findings highlight the critical interplay between retinal glial cells and the importance of Trx in modulating oxidative stress and inflammatory responses within the retinal microenvironment.

Apoptosis, is a well-regulated programmed cell death process that is controlled by the interaction of Bcl-2 family proteins. While Bcl-2 acts as a key anti-apoptotic regulator, its inhibitory effects can be counteracted by the pro-apoptotic protein Bax, thereby promoting cell death [30,31]. The caspase cascade, particularly the activation of caspase-3 by caspase-9 and caspase-8, plays a vital role in executing apoptosis. Caspase-3 is widely recognized as a reliable marker of apoptotic cell death [32]. In our study, high-glucose conditions induced significant apoptotic changes, characterized by decreased Bcl-2 and concurrent upregulation of Bax and caspase-3. Notably, treatment with Trx-enriched, conditioned media effectively reduced these effects, as evidenced by the reduced Bax and caspase-3 expression and restored Bcl-2 levels, underscoring its anti-apoptotic potential in vitro. These findings position Trx-enriched, conditioned media as a promising therapeutic strategy to combat hyperglycemia-induced apoptosis, offering new hope for cytoprotection in diabetic complications.

## 4. Materials and Methods

### 4.1. Diabetic Mouse Model Establishment

C57BL mice with established diabetes at 2, 6, and 10 weeks post-induction, weighing 20~25 g, were obtained from Dalian Medical University and acclimated for one week. To induce the diabetes model, mice were intraperitoneally injected with 50 mg/kg STZ for five consecutive days, and the normal group was intraperitoneally injected with an equal amount of sodium citrate buffer. Successful diabetes induction was confirmed by random glucose levels exceeding 16.7 mmol/L [3]. The experimental protocol was approved by the university’s animal care center.

### 4.2. Electroretinogram (ERG)

Electroretinography (ERG) was performed using an electrophysiology system (Guote Medical, V8.1 Chongqing, China). The mice were dark-adapted overnight before the experiment. The mice were deeply anesthetized by an intraperitoneal injection of pentobarbital sodium (75 mg/kg). Their pupils were dilated with 0.5% tropicamide and 0.5% phenylephrine eye drops. Once anesthetized, the mice were positioned on a detection platform after being sedated. A platinum circellus electrode was placed in contact with the central cornea and a reference electrode was inserted beneath the cheek mucosa. In this study, the amplitude of the a wave was defined as the distance from baseline to the a-wave trough, while the amplitude of the b wave was measured as the distance between the trough and peak of each waveform.

### 4.3. Morphological Analysis

The eyeball was extracted, and the upper part was labeled and fixed for 24 h with solution. Then, the eyeball was transferred to 70% ethanol for about 48 h. After being dehydrated in an ethanol gradient (80%, 90%, 95%, and 100%), xylene was used for transparency, and then the eyes were cut into 5 μm paraffin sections that contained the entire retina, including the optic disc. The slices were stained with hematoxylin and eosin. Using Element BR software (Nikon, ECLIPSE 80i, Ver5.30.00, both developed and manufactured by Nikon Corporation, a Japanese company headquartered in Tokyo), the retinal optic papilla was centered to observe the morphology of the retina.

### 4.4. TdT-Mediated dUTP Nick-End Labeling

Cells were fixed with 4% paraformaldehyde for 20 min at room temperature. A TUNEL assay was performed using the kit (KeyGEN, KGA7071, Nanjing KeyGen Biotech Co., Ltd., a biotechnology company based in Nanjing, China) according to the manufacturer’s instructions. Nuclei were counterstained with DAPI for 15 min. Images were acquired using a Leica DM6000B florescence microscope, Germany.

### 4.5. Fluorescent Immunohistochemical Staining

Cells were seeded on coverslips placed in a 24-well plate (Guangzhou Jet Bio-Filtration Co., Ltd., Guangzhou, China) and subjected to various treatments. The cells and tissues were fixed for 20 min at room temperature using a 4% formaldehyde solution. The coverslips than washed with 1% TBST and permeabilized by incubation with 0.25% Triton X-100 for 30 min at room temperature. Subsequently, the primary antibodies S-Opsin (Millipore, ABN166, 1:2000) and Iba1 (abcam, ab178846) were incubated overnight at 4 °C. After incubation, the coverslip was washed with TBST 3 times for 3 min, and then the cells were incubated with secondary antibody (Servicebio Cy3-labeled goat anti-rabbit IgG GB21303, 1:600) for 1 h at room temperature. The cells were washed 3 times for 3 min. The nuclei were counterstained with DAPI (Roche, Cat. No. 10236276001, made in Indianapolis, IN, USA) for 15 min and fluorescence images were captured using a microscope.

### 4.6. Cell Culture

The BV2 and Müller cells were cultured in Dulbecco’s modified Eagle’s medium (DMEM, Gibcoo, 4.5 g/L D-glucose) containing 10% fetal bovine serum (FBS, NEWZERUM, cat. No. FBS-UE500, a company based in Christchurch, New Zealand), 1% antibiotic–antimycotic solution (penicillin/streptomycin) at 5% CO_2_ and 37 °C in a humidified incubator. The medium was replaced every 1 or 2 days.

Once the BV2 and Müller cells reached 60–70% confluency, the cells were treated with high-glucose DMEM (50 mM) for 48 h to simulate hyperglycemic conditions. After treatment, the high-glucose DMEM was replaced with serum-free normal DMEM, and the cells were incubated for an additional 24 h. At the end of incubation, the conditioned media were centrifuged to remove cellular debris, and the resulting supernatant was collected and stored at −80 °C for the subsequent treatment of 661w cells.

The 661w cells were cultured in Dulbecco’s modified Eagle’s medium (DMEM, Gibco, 1 g/L D-glucose) containing 10% fetal bovine serum (FBS) and 1% antibiotic–antimycotic solution (penicillin/streptomycin) at 5% CO_2_ and 37 °C in a humidified incubator. Once the cells reached 70–80% confluency, the medium was replaced with Trx-enriched, conditioned media derived from BV2 and Müller cells. After 24 h of incubation, the media was removed, and the 661w cells were collected for Western blot analysis.

### 4.7. Western Blot

The cells were collected and lysed, put on ice, and an appropriate amount of cell lysate solution was used. The protein concentration in the supernatant was measured by BCA assay. Equal quantities of proteins were separated by SDS-PAGE and electro blotted on a polyvinylidene fluoride (PVDF) membrane. The membrane was blocked with 5% milk for 1 h at room temperature. After blocking, the membrane incubated with the appropriate antibodies over night at 4 °C. The membranes were incubated with anti-tubulin (proteintech, 66031-1-1 g, 1:1000), anti-O-GlcNAc (abcam, ab2739, 1:1000), anti-OGT (proteintech, 11576-2-AP, 1:1000), anti-OGA (proteintech, 14711-1-AP, 1:1000), anti-Bax (proteintech, 60267-1-1 g 1:5000), anti-Bcl2 (Abmart, T40056, 1:1000), anti-Txnip (proteintech, 18243-1-AP, 1:1000), anti-caspase3 (proteintech, 19677-1-AP, 1:500), anti-GFAP (proteintech, 16825-1-AP, 1:500) antibodies overnight at 4 °C. After washing 3 times with 1 × TBST for 15 min, the membrane was incubated with goat anti-mouse IgG (proteintech, SA, 00001-2, 1:5000) and goat anti-rabbit IgG (proteintech, SA, 00001-2, 1:5000) secondary antibodies for 1.5 h at room temperature. Again, the membrane was washed as mentioned previously, the protein bands were visualized using image lab (Version:4.1.0.2177), and the data were analyzed using image J software (Bethesda, MD, USA) (Version:2.0.0-rc-43/1.50e).

### 4.8. CO-IP Assay

The cells were lysed and incubated with the primary antibody overnight at 4 °C, and then 30 μL Protein A/G Plus-Agarose (Proteintech, Wuhan, China, Sanying, PK 10007) was added. The samples were mixed on a rocker platform at 4 °C for 4~6 h and then centrifuged at 10,000 rpm for 2 min at 4 °C. The magnetic beads were washed and resuspended in an elution buffer. Then, the samples were boiled for 5 min to release the proteins and centrifuged to remove the protein A/G beads. Finally, the complexes were tested using Western blot.

### 4.9. Transfection

The cells were seeded into a 6-well plate at a density of 2 × 10^5^ cells/mL. The Txnip-shRNA plasmid and Lipofectamine 2000 were separately diluted in low-glucose DMEM (without FBS and penicillin), gently mixed, and incubated at room temperature for 5 min. Subsequently, these two mixtures were combined and incubated at room temperature for an additional 15 min. Low-glucose DMEM containing FBS and PS (penicillin) was then added to the transfection mixture. The complex was added into each well of the plates for 24 h.

### 4.10. Statistical Analysis

GraphPad Prism 10.1.2 (LLC, San Diego, CA, USA) was used to analyze the data. The results are presented as the mean  ±  SD. The Student’s *t*-test was used to compare the means between the two groups. One-way analysis of variance followed by Dunnett’s test was used for multiple comparisons. A *p*-value less than 0.05 was considered significant.

## 5. Conclusions

In conclusion, our study highlights the critical role of dysregulated O-GlcNAc modification in the pathogenesis of DR. Importantly, we investigated the therapeutic potential of enhancing Trx within microglial and Müller cells to change the retinal microenvironment, which can mitigate photoreceptor cell damage in vitro. These findings provide a strong rationale for further research into O-GlcNAc modification and Txnip/Trx as a strong therapeutic target for the treatment of DR. Further investigations focusing on combinatorial therapeutic approaches, including antioxidant strategies and metabolic pathway modulation, may open a new avenue for preserving vision in DR patients in the future.

## Data Availability

Data are available upon request.

## Abbreviations

The following abbreviations are used in the manuscript:

DR	Diabetic retinopathy
DM	Diabetes mellitus
Trx	Thioredoxin
Txnip	Thioredoxin-interacting protein
H & E	Hematoxylin and eosin
ERG	Electroretinogram
FBS	Fetal bovine serum
GFAP	Glial fibrillary acidic protein
NVU	Neurovascular unit
ROS	Reactive oxygen species
